# Einfluss der Datenschutz-Grundverordnung auf die Datenqualität bei der Erhebung von Registerdaten: Selektionsbias aufgrund datenschutzrechtlicher Anforderungen und dessen Auswirkungen auf Kennzahlen zur Messung der Versorgungsqualität am Beispiel eines überregionalen Traumazentrums

**DOI:** 10.1007/s00113-022-01155-2

**Published:** 2022-03-11

**Authors:** Orkun Özkurtul, N. von Dercks, C. Fuchs, A. Keß, G. Osterhoff, M. F. Struck, A. Scholtz, C. Josten, J. K. M. Fakler

**Affiliations:** 1 Klinik und Poliklinik für Orthopädie, Unfallchirurgie und Plastische Chirurgie, Universitätsklinikum Leipzig AöR, Liebigstr. 24, 04103 Leipzig, Deutschland; 2 Klinik für Orthopädie und Unfallchirurgie (XIV), Bundeswehrkrankenhaus Hamburg, Lesserstr. 180, 22049 Hamburg, Deutschland; 3 Klinik und Poliklinik für Anästhesiologie und Intensivmedizin, Universitätsklinikum Leipzig AöR, Liebigstr. 24, 04103 Leipzig, Deutschland; 4 Stabsstelle Datenschutz, Universitätsklinikum Leipzig AöR, Liebigstr. 20, 04103 Leipzig, Deutschland; 5 Klinik für Unfall-, Hand-, Wiederherstellungs- und Wirbelsäulenchirurgie, Klinikum Passau, Passau, Deutschland

**Keywords:** Aufklärung, Qualitätsindikatoren, Patientenversorgung, Registerforschung, Datensicherheit, Informed consent, Data safety, Patient care, Registry research, Quality indicators

## Abstract

**Hintergrund:**

Durch die neue Datenschutz-Grundverordnung (DS-GVO) sind die Anforderungen an eine sachgerechte Aufklärung der Patienten über die Dokumentation pseudonymisierter, personenbezogener Daten in einem Register enorm gestiegen. Dies betrifft ganz besonders das TraumaRegister DGU®, da eine schriftliche Aufklärung schwer verletzter Patienten in der Akutsituation nicht immer gelingt. Ziel der Untersuchung war es, den Einfluss der infolge fehlender Aufklärung nichtdokumentierten Fälle auf die standardisierte Mortalitätsrate (SMR) zu untersuchen.

**Material und Methode:**

Im Jahr 2019 wurden 274 Patienten retrospektiv erfasst, die die Kriterien des Basiskollektivs erfüllen. Darunter waren 72 Sekundärverlegungen, die ausgeschlossen wurden. Bei den verbliebenen 197 Patienten konnte in allen Fällen der RISC II Score erhoben werden.

**Ergebnisse:**

Von den 197 primär (72 % männlich) versorgten Patienten wurden 147 (74,6 %) schriftlich aufgeklärt oder waren verstorben und wurden folglich dokumentiert. Die prognostizierte Letalität, tatsächliche Letalität und SMR betrug 18,5 %, 19,0 % resp. 1,03. Bei den Patienten, die nicht aufgeklärt wurden (*n* = 50), lagen die prognostizierte Letalität, tatsächliche Letalität und SMR bei 7,0 %, 0 % resp. 0. Bezieht man diese Fälle mit ein, ergibt sich eine deutlich günstigere SMR mit 0,93.

**Schlussfolgerung:**

Durch die fehlende schriftliche Einwilligung überlebender Patienten konnten nur etwa 75 % aller Patienten der Uniklinik Leipzig für das TraumaRegister DGU® dokumentiert werden. Da die lokale Gesetzeslage andererseits eine Registerdokumentation verstorbener Patienten zulässt, ergibt sich daraus eine nachteilige Beeinflussung der SMR, die in unserem Kollektiv etwa 10 % höher ausfällt, als sie in Realität ist.

## Abstract (en)

**Background:**

Due to the new general data protection regulations (GDPR), the requirements for correct patient information on the documentation of pseudonymized data in a registry have increased enormously. In particular, written consent applies to the TraumaRegister DGU® as it is not always possible to get written permission from severely injured patients in acute situations. Therefore, the study aimed to investigate the influence of undocumented cases due to a lack of clarification on the standardized mortality rate (SMR).

**Material and methods:**

In 2019, 274 patients meeting the criteria of the baseline dataset were retrospectively recorded. In the remaining 197 patients, the RISC II score could be calculated in all cases. In addition, due to state-specific law, all deceased patients were documented in our trauma center.

**Results:**

In this study with 197 primary care patients (72% male), 147 (74,6%) were informed and gave permission or died and were subsequently documented. The predicted mortality, actual mortality and SMR were 18.5%, 19.0% and 1.03, respectively. For patients who were not informed (*n* = 50), the predicted mortality, actual mortality, and SMR were 7.0%, 0% and 0. When these cases are included, the SMR is significantly more favorable at 0.93.

**Conclusion:**

Due to the lack of written consent from surviving patients, only about 75% of all patients at Leipzig University Hospital could be documented for the TraumaRegister DGU®. On the other hand, since the local legal situation permits registry documentation of deceased patients, this has a detrimental effect on the standardized mortality rate (SMR), which is about 10% higher in our collective than it actually is.

## Einleitung

Nach wie vor ist die Polytraumatisierung die Todesursache Nummer eins bei Patienten unter 40 Jahren [1]. Seit über 25 Jahren werden schwer verletzte Patienten in Deutschland im Traumaregister der Deutschen Gesellschaft für Unfallchirurgie (DGU) e. V. (TraumaRegister DGU®, TR-DGU) zum Zwecke der externen Qualitätssicherung und für wissenschaftliche Zwecke erfasst. Der Standardbogen zur Datenerhebung umfasst im Mittel 212 Variablen zu den unterschiedlichen prä- und innerklinischen Versorgungsphasen der Schwerverletztenversorgung. Am TR-DGU nehmen bundesweit über 600 Traumazentren teil und dokumentieren pro Jahr ca. 30.000 Datensätze im TR-DGU [6, 22]. Diese werden zur Darstellung der Versorgungsrealität herangezogen und bilden die Versorgungsqualität in den entsprechenden Versorgungsebenen ab [20]. Hierfür ist es unabdingbar, die insbesondere hoch relevanten Qualitätsindikatoren für die Berechnung und Erfassung der prognostizierten und tatsächlichen Letalität zu erfassen. Die prognostizierte Letalität wird mittels Revised Injury Severity Classification II (RISC II) Score ermittelt [16]. Als Minimalanforderung für diesen Score gelten das Alter des Patienten sowie das Verletzungsmuster. Um Patienten mit unterschiedlichen Verletzungen anhand der Prognose und Letalität vergleichen zu können, wurde die „standardized mortality ratio“ (SMR) eingeführt [10]. Dieser gilt in den jeweiligen Traumanetzwerken als Qualitätsindikator und wird als Benchmark für die jeweilige Klinik hinsichtlich der Prozessqualität relevant. Die SMR ist der Quotient aus beobachteter und prognostizierter Mortalität. Dabei wird ein Wert ermittelt, der im Idealfall 1 beträgt. Fällt der Wert höher aus, ist auch die Letalität höher; fällt er niedriger aus, ist die Letalität geringer als prognostiziert. Eine niedrige SMR ist erstrebenswert, weil sie für ein günstigeres Behandlungsergebnis spricht, als man erwarten konnte. Eine hohe SMR sollte Anlass geben, nach Verbesserungsmöglichkeiten der Versorgung zu suchen, weil sie Ausdruck eines ungünstigen Behandlungsergebnisses ist, bei dem mehr Patienten verstorben sind als erwartet werden konnte. Die Qualität und Vollständigkeit der Datenerhebung ist wegen der daraus errechneten SMR daher essenzieller Bestandteil der Qualitätssicherung der bundesweiten Versorgung polytraumatisierter Patienten [14]. Die rechtlichen Rahmenbedingungen haben sich mit der Einführung der DS-GVO im Mai 2018 gewandelt. Die drohenden Sanktionen aus den Bedingungen der DS-GVO erfordern erhebliche Anstrengungen für die Einhaltung datenschutzrechtlicher Bestimmungen bei der Verarbeitung medizinischer Daten [22, 23]. Um eine lückenlose Dokumentation in das TraumaRegister DGU® zu gewährleisten, sind eine hinreichend informierte Einwilligung nebst Aufklärung aller schwer verletzten Patienten und die Beachtung hochdifferenzierter Regelungen zur Datensicherheit erforderlich. Dies ist aus pragmatischen und medikolegalen Gründen nicht immer zu leisten. Eine Aufklärung kann beispielsweise dann nicht erfolgen, wenn der Patient intubiert wurde oder wegen seiner Verletzungen auch nicht geschäftsfähig wäre. Zudem lehnen einige Patienten die Weitergabe und Verarbeitung ihrer Daten, trotz ausführlicher Aufklärung, ab.

Problematisch ist auch, dass die Gesetze zum Datenschutz auf Ebene der Bundesländer unterschiedlich ausgestaltet sind. Während sich der Datenschutz in Hamburg über den Tod hinaus erstreckt, ist dies in anderen Bundesländern nicht der Fall (§7 Abs. 1 S. 3 HmbKHG, www.lexsoft.de). Dies kann zu Verzerrungen bei der Dokumentation und Interpretation von Sterblichkeitsraten und damit zusammenhängenden Qualitätsindikatoren führen. Erst kürzlich wurde vom scheidenden Präsidenten der DGU, Prof. Raschke, darauf hingewiesen, dass es zu einem Rückgang der Fälle seit Einführung der DS-GVO 2018 von zunächst 6 % und 2019 von 17 % gekommen ist [6].

Ziel dieser Arbeit war es, den Einfluss überlebender, mangels Einwilligung nicht im TraumaRegister DGU® dokumentierter Patienten auf die Qualitätsindikatoren tatsächliche Mortalität sowie die SMR an einem überregionalen Traumazentrum zu analysieren und hinsichtlich eines möglichen Selektionsbias zu bewerten.

## Material und Methoden

In einem überregionalen Traumazentrum wurde im Rahmen einer retrospektiven, monozentrischen Studie das Kollektiv von Schockraumpatienten des Jahres 2019 evaluiert. Die Patienten, die die Kriterien des Basiskollektivs des TraumaRegister DGU® erfüllten, wurden eingeschlossen. Zuverlegte Patienten sowie Patienten, die primär wegen ihrer moribunden Grunderkrankungen verstorben sind, wurden ausgeschlossen. Während des stationären Verlaufs erfolgten die Aufklärung und Einholung der Einwilligung zur Dokumentation ins TraumaRegister DGU®. Bei Zustimmung erfolgte eine vollständige Dokumentation dieser Patienten. Die Daten dieser Patienten wurden pseudonymisiert an das TraumaRegister DGU® übermittelt und standen gleichzeitig für eine klinikinterne Auswertung zur Verfügung. In Fällen, in denen keine Einwilligung vorlag, wurde lediglich ein klinikinterner Datensatz angelegt; eine Weiterleitung in das Register erfolgte nicht. Eine Ausnahme stellten die verstorbenen Patienten dar, deren Daten auch ohne eine vorliegende Einwilligung im TraumaRegister DGU® dokumentiert wurden. Aufgrund landesspezifischer Regelungen in Sachsen greift der Datenschutz unter bestimmten Bedingungen nicht über den Tod hinaus, weshalb personenbezogene Daten dann im TraumaRegister DGU® hinterlegt werden können. Eine rein klinikinterne Dokumentation und Verarbeitung von personenbezogenen Daten ist entsprechend des §34 Abs. 1 des Sächsischen Krankenhausgesetzes für eigene wissenschaftlichen Auswertung auch für lebende Patienten ohne Datenschutzeinwilligung möglich. Dort heißt es im Wortlaut „Ärzte dürfen Patientendaten, die innerhalb ihrer Fachabteilung oder bei Hochschulen innerhalb ihrer medizinischen Einrichtungen, in den Universitätsklinika oder in sonstigen medizinischen Einrichtungen gespeichert sind, für eigene wissenschaftliche Forschungsvorhaben verarbeiten.“ Die retrospektive Auswertung der eigenen TraumaRegister DGU®-Daten wurde durch die Ethikkommission der Universität Leipzig geprüft (AZ 427/18-ek).

Die vorliegende Auswertung wurde mit SPSS (Version 25, IBM Inc., Chicago, IL, USA) angefertigt. Die Angaben erfolgen als Mittelwerte ± Standardabweichung (SD), bei nichtnormalverteilten Daten werden Median und interquartile Ränge (IQR) verwendet, sofern nicht anders angegeben. Der Chi-Quadrat-Test wurde im Falle von kategorisierten Daten durchgeführt. Bei stetigen Variablen, welche von der Normverteilung abwichen, erfolgte eine Analyse mit einem parameterfreien Test (Mann-Whitney-U-Test). Ein *p* < 0,05 wurde als statistisch signifikant gewertet.

## Ergebnisse

**Figure 1:**
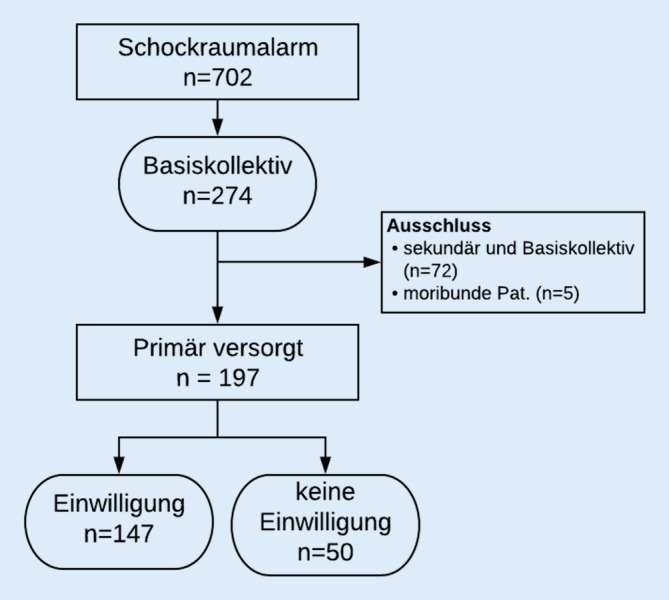

Im Jahr 2019 wurde der chirurgische Schockraum 702-mal aktiviert, 274 Patienten entsprachen den Kriterien des Basiskollektivs des TraumaRegister DGU®. Darunter waren 72 Sekundärverlegungen, die ausgeschlossen wurden. Weiterhin wurden 5 Patienten ausgeschlossen, die bereits vor dem Unfall moribund waren und infolge ihrer Grunderkrankung verstarben (Abb. 1). Bei den verbliebenen 197 primär versorgten Patienten konnte in allen Fällen der RISC II Score erhoben werden. Von diesen wurden 147 (74,6 %) schriftlich aufgeklärt oder waren verstorben und wurden folglich im TraumaRegister DGU® dokumentiert (Tab. 1). Die prognostizierte Letalität, tatsächliche Letalität und SMR betrugen 18,5 %, 19,0 % resp. 1,03. Bei den Patienten, bei denen keine Einwilligung für eine Weiterleitung ihrer Daten an das TraumaRegister DGU® vorlag (*n* = 50), wurden eine prognostizierte Letalität, tatsächliche Letalität und SMR bei 7,0 %, 0,0 % resp. 0,0 ermittelt. Bezieht man auch die Fälle ohne vorliegende schriftliche Einwilligung mit ein, ergibt sich für das Gesamtkollektiv eine deutlich günstigere SMR mit 0,93 (prognostizierte Letalität 15,2 %, tatsächliche Letalität 14,2 %). Die Patienten ohne Einwilligung waren bei vergleichbarer Verletzungsschwere (ISS 16 vs. 17) und einem vergleichbaren RISC II Score von 1,4 vs. 1,6 im Median knapp 20 Jahre jünger. Gründe für eine nichtvorliegende Aufklärung waren Ablehnung durch den Patienten (22 %), sprachliche Barrieren (20 %), unklares Betreuungsverhältnis (14 %) sowie organisatorische Gründe (44 %).

**Table 1:** 

–	Mit Aufklärung (*n* = 147)	Ohne Aufklärung (*n* = 50)	*p*-Wert
Alter	55,0 (38,8–73,0)	36,0 (22,0–55,3)	< 0,001
Geschlecht männl.	72,8 %	70 %	0,718
ASA	2,0 (1,0–2,0)	1,0 (1,0–2,0)	0,163
ASA1	48,2 %	58,3 %	–
ASA2	33,3 %	31,3 %	–
ASA3	17,7 %	10,4 %	–
ASA4	0,7 %	0,0 %	–
RRsys b. Aufn.	133,5 (111,8–150,3)	139,0 (121,8–150,0)	0,287
HFr bei Aufn	85,0 (75,0–100,0)	92,0 (78,5–99,5)	0,081
GCS bei Aufn.	15,0 (3,0–15,0)	15,0 (8,0–15,0)	0,770
Hb bei Aufn.	13,1 (11,6–15,6)	13,5 (12,7–14,6)	0,118
ISS	17,0 (11,0–27,0)	16,0 (10,0–25,0)	0,191
ISS ≥ 16	59,2 %	56,0 %	0,741
ISS < 9 (+ ICU)	1,0 %	4,0 %	0,063
RISC II	1,6 (0,5–18,2)	1,4 (0,5–6,0)	0,321
Letalität	19,0 %	0 %	< 0,001
SMR	1,03	*n*/a	*n*/a

## Diskussion

In der vorliegenden Arbeit konnte der Einfluss der DS-GVO auf die registerbezogene TR-DGU-Outcome-Analyse mittels prognostizierter und tatsächlicher Letalität sowie der SMR für ein überregionales Traumazentrum gezeigt werden. Von 50 Patienten (25,4 %) mit einem durchschnittlichen ISS von 16 (10 bis 25) Punkten konnte aus oben genannten Gründen (Ablehnung der Einwilligung, Sprachbarriere, ungeklärtes Betreuungsverhältnis) keine Einwilligungen eingeholt werden, 74,6 % gaben ihre Einwilligung oder wurden nach Versterben dokumentiert. Dies führte dazu, dass die Letalität sowie die SMR des Gesamtkollektivs mit 19 % resp. 1,03 deutlich negativer ausfielen als unter Berücksichtigung der nichteinwilligungswilligen bzw. -fähigen Patienten mit einer errechneten Gesamtletalität und SMR von 14,2 % resp. 0,93. Alter hat einen wesentlichen Einfluss auf die RISC-Prognose. Das mediane Alter der beiden Gruppen ist sehr unterschiedlich verteilt, woraus sich ein Bias ergibt. In unserer Studie lagen die Gründe für ein Fehlen der Einwilligung insbesondere bei jüngeren Patienten v. a. in Sprachbarrieren, genereller Ablehnung bzw. fehlender Motivation zu einer Teilnahme und einem Alter unter 18 Jahren, da bei diesen oft keine Einwilligung von (beiden) sorgeberechtigten Elternteilen eingeholt wurde. Die Conditiones sine quibus non bei der Motivation zur Teilnahme an Registererfassungen sind vergleichbar mit denen klinischer Studien und erfordern eine ausführliche sowie zeitintensive Aufklärung [9, 13, 15, 17]. Die niederländische Arbeitsgruppe um Nievaard et al. haben bereits 2004 eine systematische Literaturrecherche zur Teilnahme von Patienten an klinischen Studien publiziert. Auch hier konnte gezeigt werden, dass der selbstbestimmte informierte Patient eine Einwilligung erteilt, wenn der Nutzen für das Gemeinwohl und zur wissenschaftlichen Erkenntnis in ausführlichen Gesprächen klar wird [18]. Die dafür erforderlichen personellen Ressourcen können jedoch mitunter nicht immer gewährleistet werden, zumal dies im deutschen DRG-System in keiner Weise abgebildet ist [24].

Durch die TraumaRegister-DGU®-Daten werden derzeit im Mittel 212 Variablen mit dem Standardbogen erfasst, woraus u. a. 8 Qualitätsindikatoren zur Bewertung der prä- und innerklinischen Behandlungsergebnisse abgeleitet werden [14, 15, 22]. Für eine verlässliche Betrachtung des Behandlungsergebnisses ist eine unverzerrte und repräsentative Stichprobe erforderlich. Durch den in dieser Studie aufgezeigten Selektionsbias wird die im Sozialgesetzbuch V (SGB V) festgeschriebene Forderung nach einem systematischen, externen Qualitätsmanagement im Gesundheitswesen konterkariert.

Auch in rechtlicher Hinsicht ist die Einwilligung an sich als Instrument der Legitimation im geschilderten Kontext zu diskutieren, da sie unter bestimmten, ungünstigen Umständen letztendlich ebenso dem gesetzlichen Auftrag zur externen Qualitätssicherung entgegenstehen kann. Denn eine Einwilligung ist an verschiedene Voraussetzungen gebunden, damit diese als rechtskonform und damit überhaupt als wirksam angesehen werden kann. Ebenso muss sich der Verantwortliche dahingehend exkulpieren können, dass ein Vollzugsdefizit beim Umgang mit der Einwilligung, im Sinne der Nachweispflicht nach Art. 5 Abs. 2 DS-GVO, zum Zeitpunkt des Einsatzes ausgeschlossen war [11, 21]. Als eine der Wirksamkeitsvoraussetzungen muss das Einwilligungsersuchen dem Transparenzgebot folgen, sodass sich der Inhalt der Einwilligung klar und leicht verständlich für den Betroffenen erschließen kann [19]. Sprachbarrieren oder der Gesundheitszustand könnten dazu führen, dass diesem Gebot nicht Folge geleistet werden kann. Spätestens im Rahmen einer Revision durch eine Aufsichtsbehörde oder im Falle einer gerichtlichen Auseinandersetzung könnte die Wirksamkeit in bestimmten Einzelfällen u. U. bezweifelt werden, wenn über das Zustandekommen beschieden werden müsste. Hinterfragt werden könnte theoretisch außerdem, ob eine Einwilligung, wie vorgeschrieben, freiwillig abgegeben werden kann, oder ob sich der Patient aufgrund des Umstands einem Druck unterworfen fühlt oder der Eindruck „eines Ungleichgewichts der Macht“ zwischen dem Patienten und der helfenden Einrichtung bzw. dem Arzt entsteht. Unter Berücksichtigung der zuvor aufgeworfenen Problematiken muss daher die Frage gestellt werden, ob die Einwilligung in Bezug auf die Arbeit in Verbindung mit nicht gesetzlich geregelten Registern von großem Interesse der Allgemeinheit sinnhaft ist, da die Rechtswirksamkeitsvoraussetzungen im ungünstigsten Fall unterminiert werden könnte [2, 8], oder ob mitgliedstaatliche Rechtsinstrumente wie z. B. modifizierte Datenschutzregularien für die Registerarbeit förderlicher wären. In der gelebten Praxis stellt sich ohnehin die Frage, ob sich Patienten mit einem derzeit 8 Seiten langen Informations- und Einwilligungsbogen für die Weiterleitung pseudonymisierter Daten an das TraumaRegister DGU® umfänglich auseinandersetzen, nachdem sie aufgrund eines akuten Unfallereignisses aus ihrem alltäglichen Leben herausgerissen und nicht selten mit drohender Invalidität und existenziellen Sorgen beschäftigt sind. Eine Einwilligung zu einem späteren Zeitpunkt, ggf. sogar nach der akutstationären Behandlung, wäre denkbar und möglich, allerdings zeigt unsere Erfahrung, dass hier die Rücklaufquote bestenfalls 25 % beträgt.

Ein möglicher Lösungsansatz wäre, die pseudonymisierte Datenakquise technisch so zu verschlüsseln, dass eine DS-GVO-konforme Datenschutzumgebung gewährleistet und so eine Datenanalyse ohne Einwilligung möglich gemacht wird [15]. Allerdings ist diese technische Umsetzung durch die Diversität der Klinikstrukturen sowie unterschiedliche, dem Föderalismus geschuldete gesetzliche Regelungen bis heute nicht realisierbar.

Durch die landesspezifischen Regelungen kommt es bei der Dokumentation in das TraumaRegister DGU® zu der sehr speziellen Situation, dass manche Kliniken theoretisch nur noch Daten verstorbener Polytraumapatienten in das Register eingeben könnten, wenn alle anderen die Einwilligung zur Datenweitergabe ablehnten [7]. Gleichwohl gibt es auch Bundeländer, in denen der Datenschutz über den Tod hinaus Bestand hat und hier verstorbene Patienten, die vor ihrem Ableben keine Einwilligung abgegeben haben, nicht im Register erfasst werden können. So gibt es beispielsweise in Sachsen eine andere Regelung als in Hamburg. In Sachsen greift der bereichsspezifische Datenschutz nach §35 Abs. 5 SGB I, wonach die Sozialdaten Verstorbener verarbeitet werden dürfen, „wenn schutzwürdige Interessen des Verstorbenen oder seiner Angehöriger dadurch nicht beeinträchtigt werden können“. Diese Regelungen wurden in unterschiedlicher Form den politischen Entscheidungsträgern kommuniziert und darauf hingewiesen [4, 7]. Eine gesetzliche Regelung mit verpflichtender Teilnahme würde Rechtssicherheit schaffen und die Daten deutlich aufwerten [7]. Diese verzerrende Entwicklung durch landesdatenschutzspezifische Regelungen lässt sich auf alle teilnehmenden Kliniken des TraumaRegister DGU® anwenden [5, 12].

Die EU hat in ihrer Richtlinie personenbezogene Daten verstorbener Personen aus dem Geltungsbereich der DS-GVO vollständig und bewusst herausgenommen und eine Öffnungsklausel für die Verarbeitung dieser Daten bereitgehalten. Von dieser Öffnungsklausel hat der deutsche Gesetzgeber bisher keinen Gebrauch für eine Regel im Bundesdatenschutzgesetz gemacht.

In einer kleinen Anfrage an die Bundesregierung zur Zukunft des deutschen Traumaregisters (Deutscher Bundestag, Drucksache 19/30638) wurde seitens der Bundesregierung festgestellt, dass es einer „umfassenden Prüfung“ bedürfe, „ob und inwieweit gesetzlicher Anpassungsbedarf für das Traumaregister“ bestehe und zunächst eine Lösung „auf Basis des bestehenden Rechts“ zu prüfen sei. Eine Gesetzesinitiative sei „derzeit seitens der Bundesregierung nicht beabsichtigt“. Dies ist jedoch angesichts des hier dargestellten Selektionsbias geboten, um den Bedürfnissen der Registerforschung gerecht werden zu können und die auf die DS-GVO zurückgeführte Verzerrung des Behandlungsergebnisses – wie hier am Beispiel des Universitätsklinikums Leipzig gezeigt – gerecht zu werden [3].

## Limitationen

Es handelt sich um eine retrospektive „Single-center“-Studie mit den sich daraus ergebenden Limitationen. Insgesamt ist die Fallzahl relativ gering und die Analyse gegenüber statistischen Ausreißern anfällig.

## Schlussfolgerung

Durch die DS-GVO wurde an unserem Traumazentrum wegen fehlender schriftlicher Patienteneinwilligungen etwa ein Viertel weniger Patienten im TraumaRegister® dokumentiert. Da die lokale Gesetzeslage jedoch eine Registerdokumentation verstorbener Patienten auch ohne schriftliche Einwilligung erlaubt, ergibt sich an unserem Traumazentrum ein deutlich negativer Bias mit ungünstiger ausfallender SMR. Der Gesetzgeber muss Regelungen schaffen, die den Bedürfnissen der Registerforschung gerecht werden, denn immerhin führen Probleme, die auf die DS-GVO zurückgeführt werden, zu einer erheblichen Verzerrung des Behandlungsergebnisses, wie in unserer Studie gezeigt werden konnte.

## Abkürzungen

ASA: American Society of Anesthesiologists (Risikoklassifikation von operativen Patienten der American Society of Anesthesiologists zur Einteilung des Gesundheitszustands)
GCS: Glasgow Coma Scale
HF: Herzfrequenz
ICU: Intensive care unit
ISS: Injury Severity Score
RISC II: Revised Injury Severity Classification Score II
RRsys: Systolischer Blutdruck nach Riva Rocci
SMR: Standardisierte Mortalitätsrate
TR-DGU®: Traumaregister® der Deutschen Gesellschaft für Unfallchirurgie
